
JOURNAL INFORMATION
==============================
NLM Title Abbreviation: Biospektrum (Heidelb)
Journal ID: Biospektrum (Heidelb)
NLM Journal ID: 9615685

Biospektrum

ISSN: 0947-0867
EISSN: 1868-6249

ARTICLE INFORMATION
==============================
PMCID: PMC7880639
PMID: 33612988
DOI: 10.1007/s12268-021-1535-3
Article ID: 1535
Article version: 1
Subjects: Wissenschaft

Den molekularen Wirtszellveränderungen durch SARS-CoV-2 auf der Spur

Klann Kevin 1
Münch Christian ch.muench@em.uni-frankfurt.de
1 2 3
1 Institut Biochemie II, Universitätsklinikum, Universität Frankfurt a. M., Theodor-Stern-Kai 7, D-60590 Frankfurt a. M., Deutschland
2 Frankfurt Cancer Institute, Frankfurt A. M., Deutschland
3 Cardio-Pulmonary Institute, Frankfurt A. M., Deutschland
Electronic publication date: 2021 Feb 12
Print publication date: 2021
Volume: 27
Issue: 1
First page: 40
Last page: 45
Copyright: © Die Autoren 2021
License: Dieser Artikel wird unter der Creative Commons Namensnennung 4.0 International Lizenz veröffentlicht, welche die Nutzung, Vervielfältigung, Bearbeitung, Verbreitung und Wiedergabe in jeglichem Medium und Format erlaubt, sofern Sie den/die ursprünglichen Autor(en) und die Quelle ordnungsgemäß nennen, einen Link zur Creative Commons Lizenz beifügen und angeben, ob Änderungen vorgenommen wurden. Die in diesem Artikel enthaltenen Bilder und sonstiges Drittmaterial unterliegen ebenfalls der genannten Creative Commons Lizenz, sofern sich aus der Abbildungslegende nichts anderes ergibt. Sofern das betreffende Material nicht unter der genannten Creative Commons Lizenz steht und die betreffende Handlung nicht nach gesetzlichen Vorschriften erlaubt ist, ist für die oben aufgeführten Weiterverwendungen des Materials die Einwilligung des jeweiligen Rechteinhabers einzuholen. Weitere Details zur Lizenz entnehmen Sie bitte der Lizenzinformation auf http://creativecommons.org/licenses/by/4.0/deed.de.
License URL: https://creativecommons.org/licenses/by/4.0/

issue-copyright-statement: © Springer-Verlag GmbH Deutschland, ein Teil von Springer Nature 2021

==============================
Upon infection with SARS-CoV-2, a variety of changes happen inside the host cell. The virus hijacks host cell pathways for driving its own replication, while the host counteracts with response mechanisms. To gain a comprehensive understanding of COVID-19, caused by SARS-CoV-2 infection, and develop therapeutic strategies, it is crucial to observe these systematic changes in their entirety. In our recent studies, we followed the effects of SARS-CoV-2 infection on the human proteome, which led to the identification of several drugs that abolished viral proliferation in cells.

■ Der Befall einer Wirtszelle mit Viren löst unterschiedlichste Änderungen innerhalb der Zelle aus. Während das Virus bestrebt ist, sich selbst und sein Erbgut zu vermehren, versucht die Wirtszelle, dies zu verhindern. Es entsteht ein regelrechtes Tauziehen um die Hoheit über den zellulären Stoffwechsel. Die Proteinsynthesemaschinerie ist ein typisches Beispiel: Verschiedene Viren versuchen diese auf die Produktion viraler Proteine umzustellen [1] und die Wirtszelle reagiert mit einer Abschaltung der Proteinbiosynthese [2]. Umgekehrt versucht die Wirtszelle das Immunsystem zu aktivieren, während das Virus dieses blockiert. Dies sind nur zwei Beispiele für den erbitterten Wettkampf zwischen Wirt und Virus auf zellulärer Ebene. Insgesamt greift eine virale Infektion in der Regel massiv in die

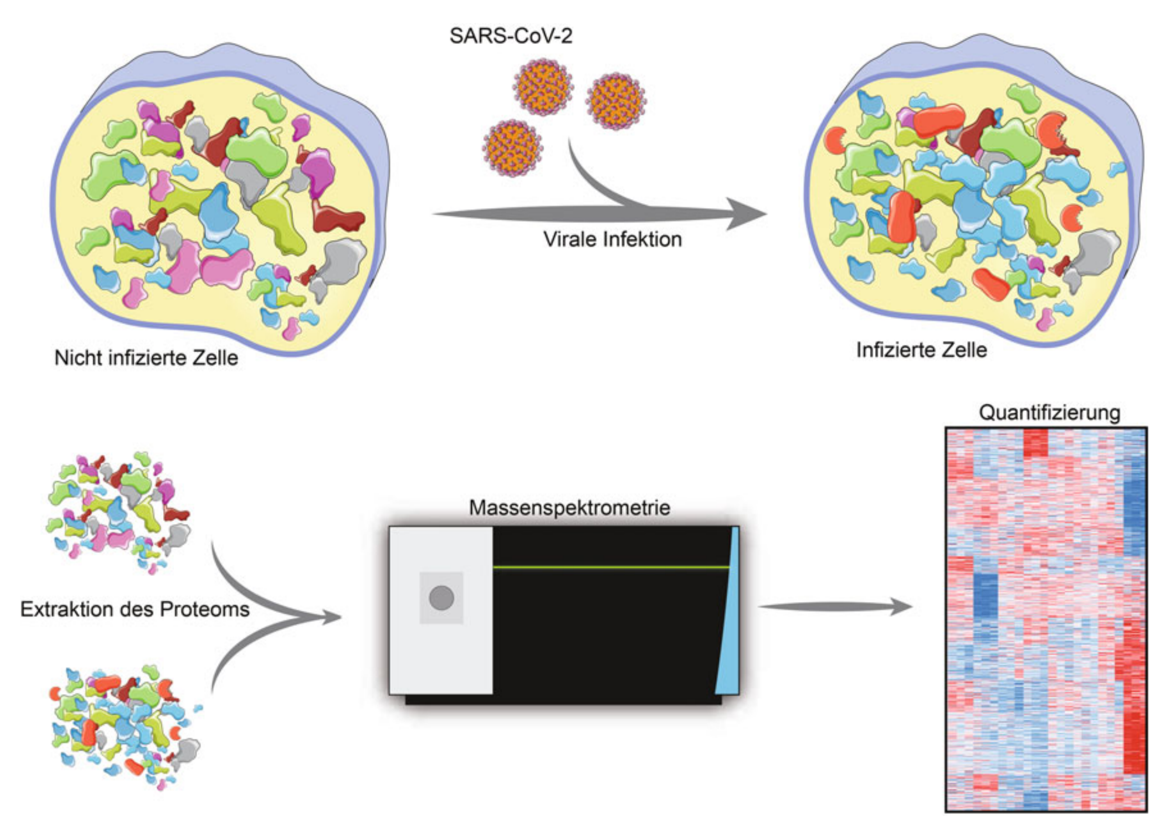

◄ **Abb. 1:** Schema der Proteomanalyse mittels Massenspektrometrie. Durch die Infektion mit dem SARS-CoV-2-Virus kommt es zu globalen Veränderungen in der Proteinzusammensetzung der Zelle. Wirtsproteine werden abgebaut oder verstärkt gebildet und virale Proteine synthetisiert. Die Extraktion des kompletten Proteoms der Zellen vor und nach Infektion ermöglicht die Quantifizierung einzelner Komponenten in einem modernen Massenspektrometer.

Hier steht eine Anzeige.

Springer

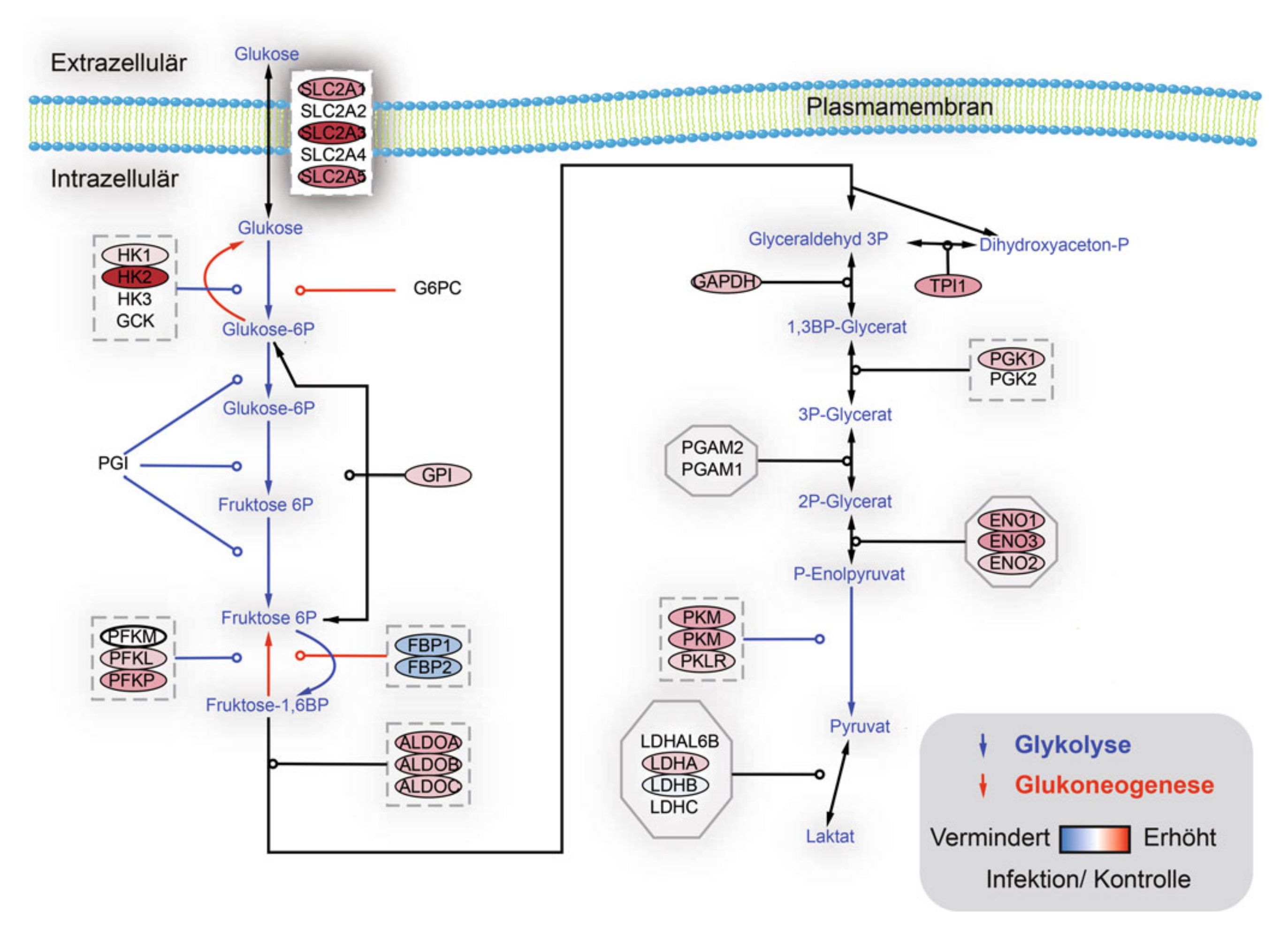

▲ **Abb. 2:** Schematische Darstellung des Glukosestoffwechselwegs nach Infektion mit SARS-CoV-2. Die Abbildung wurde auf Basis der WikiPathways-Datenbank erstellt und modifiziert. Zu sehen sind die wichtigsten Enzyme des Stoffwechselwegs und die jeweiligen Stoffwechselzwischenprodukte. Die Proteine sind, soweit detektiert, nach ihrer Veränderung im Proteinlevel eingefärbt. Rot bedeutet eine Erhöhung des Proteinlevels, blau eine Verminderung.

Synthese, die Modifikation und den Abbau von Proteinen ein. Welche Änderungen ein Virus in der Zelle dabei auslöst, kann von Virus zu Virus stark variieren. Daher ist die genaue Untersuchung dieser Vorgänge essenziell, um die dadurch verursachten Erkrankungen zu verstehen und geeignete Therapieansätze zu entwickeln. Durch die medikamentöse Unterstützung der Wirtszelle beim molekularen Tauziehen kann das Virus ausgebremst oder sogar vollständig blockiert werden.

Hier steht eine Anzeige.

Springer

Nach dem Ausbruch des Coronavirus *severe acute respiratory syndrome coronavirus 2* (SARS-CoV-2) und dem Auftreten erster Fälle unter Rückreisenden aus China ist es unseren Kollegen von der Medizinischen Virologie am Universitätsklinikum Frankfurt (Jindrich Cinatl und Sandra Ciesek) frühzeitig gelungen, das Virus zu isolieren [3]. Mit dem gewonnenen Virusisolat konnten humane Zellen im Labor infiziert werden, sodass wir schon bald die Auswirkungen einer SARS-CoV-2-Infektion auf Wirtszellen untersuchen konnten [4, 5]. Dafür verwendeten wir moderne massenspektrometrische Analysemethoden, die es erlauben, einen Großteil der Proteine einer Zelle (das Proteom) zeitgleich und mit hoher Messgenauigkeit zu untersuchen (**Abb. 1**).

### Wie misst man die Vielzahl von Veränderungen im Proteom?

Das humane Genom codiert für über 20.000 verschiedene Genprodukte, von denen zumindest 13.000–14.000 gleichzeitig in einem Zelltyp produziert werden. Während die klassische Herangehensweise des Western Blots einzelne Proteine durch Antikörperreaktionen nachweist, ermöglichen es moderne Massenspektrometer, einen großen Teil des Proteoms in einem einzelnen Experiment zu quantifizieren und charakterisieren. Große Fortschritte in den vergangenen Jahren haben dazu geführt, dass nicht nur Proteinmodifikationen wie Phosphorylierungen oder Ubiquitinierungen, sondern auch dynamische Änderungen von Proteinen quantitativ erfasst werden können [4]. Dabei werden neu gebildete Proteine metabolisch mit Isotopen markiert, welche sich dann in ihrer Masse von den bereits existierenden Proteinen abheben und somit messen lassen [6]. Dadurch kann die Regulation der Proteinsynthese und des -abbaus unter

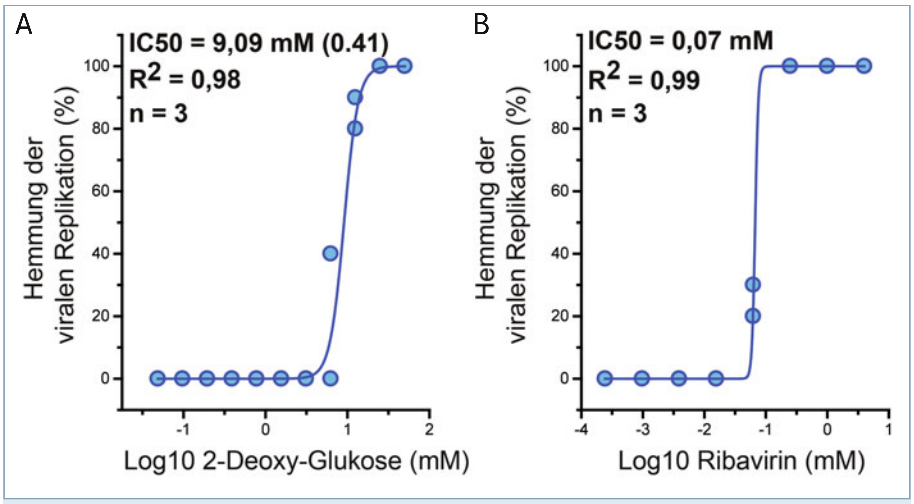

▲ **Abb. 3:** Inhibition der viralen Replikation durch Hemmung relevanter Stoffwechselwege. **A**, Die Glykolyse wurde durch den Stoff 2-Deoxyglucose gehemmt und blockiert somit die virale Replikation. 2-Deoxyglucose befindet sich zurzeit in der Evaluation als Medikament gegen Tumorerkrankungen. **B**, Außerdem konnte mit dem bereits für andere Viruserkrankungen zugelassenen Medikament Ribavirin ein ähnliches Ergebnis erzielt werden. Die Hemmung des Enzyms IMPDH führt ebenfalls zur Blockade der viralen Vermehrung. Dargestellt ist die Hemmung der viralen Replikation (in Prozent) abhängig von der verwendeten Konzentration des Medikaments. (Abbildung aus [4])

Hier steht
eine Anzeige.

Springer verschiedenen Bedingungen analysiert werden, z. B. im Kontext einer viralen Infektion [7].

### Molekulare Spurensuche im komplexen Proteinnetzwerk der Zelle

Die durch solche Experimente generierte Datenmenge ist Fluch und Segen zugleich. Zwar ist es möglich, vielschichtige Informationen von tausenden Proteinen gleichzeitig zu erhalten, aufgrund der schieren Flut an Daten gleicht dies jedoch oft der Suche nach der sprichwörtlichen Nadel im Heuhaufen – zumal sich unser Bild von der Steuerung zellulärer Funktionen und Signalwege in den vergangenen Jahrzehnten gewandelt hat. Das klassische Konzept, nach dem ein Genprodukt eine Funktion erfüllt (one-gene-one-function), war zwar ein Meilenstein der Lebenswissenschaften [8], allerdings wissen wir heute, dass die Sachlage deutlich komplexer ist. Abhängig von der Lokalisation, dem Zelltyp oder den allgemeinen Umständen können Proteine verschiedene Funktionen erfüllen. Das Netzwerk an Proteinen und Funktionen innerhalb der Zelle, in dem Komponenten sich gegenseitig beeinflussen, aktivieren oder inhibieren, ist daher äußerst komplex [9, 10]. Signalwege kreuzen sich, heben sich auf oder verstärken eine bestimmte Antwort. Daher ist es oft zielführender, die Änderungen innerhalb der Zelle nicht nur auf der Ebene einzelner Proteine zu betrachten, sondern diese in ihren funktionellen Kontext zu setzen. Diese Herangehensweise wird allgemein als Systembiologie oder, im klinischen Kontext, als Systemmedizin bezeichnet. Zeigen andere Proteine desselben Stoffwechselwegs eine ähnliche Tendenz? Welche Auswirkungen könnten Veränderungen an einer Stelle des Netzwerks auf andere Knotenpunkte haben? Diese Fragestellungen helfen dabei, die Ergebnisse in einem größeren Gesamtbild zu betrachten und die kritischen Stellen im Netzwerk, welche medikamentös gesteuert werden können, zu erkennen [11].

Hier steht eine Anzeige.
Springer

### SARS-CoV-2 moduliert den Metabolismus der Wirtszelle

Um eben jenes Gesamtbild zu untersuchen, haben wir eine menschliche Zelllinie mit SARS-CoV-2 infiziert und anschließend Wirtszell- und Virusproteine auf Veränderungen bei der Synthese, der Gesamtmenge und beim Phosphorylierungsstatus analysiert [4, 5]. So konnten wir therapeutische Ansatzpunkte aufdecken. Am stärksten waren metabolische Stoffwechselwege innerhalb der Wirtszelle von Veränderungen betroffen. Am prominentesten stach die Glykolyse hervor, ein Stoffwechselweg, der aus Zucker Energie und diverse Zwischenprodukte generiert (**Abb. 2**). Wir beobachteten, dass Enzyme der Glykolyse nicht nur in Bezug auf die Proteinmenge, sondern auch hinsichtlich des Phosphorylierungsstatus verändert waren, was auf eine wichtige Rolle dieser Enzyme während der Infektion hindeutet. Wirkstoffe, die in die Glykolyse eingreifen, gibt es bereits – diese werden in klinischen Studien für onkologische Anwendungen untersucht, da einige Krebsentitäten stark auf diesen Zweig des Stoffwechsels angewiesen sind. So lag es nahe, dieselben Präparate auf ihre Tauglichkeit gegen SARS-CoV-2 zu testen. Unsere Zellkulturexperimente zeigten, dass eine der Substanzen, 2-Deoxyglucose, in der Tat wirksam gegen die Vermehrung des Virus war (**Abb. 3A**).

Darüber hinaus wurden wir durch unsere Experimente auf ein zweites, bereits für andere Viruserkrankungen zugelassenes, Medikament namens Ribavirin aufmerksam [12, 13]. Unsere Ergebnisse zeigten, dass der Stoffwechselweg, der durch Ribavirin gehemmt wird, sich nach SARS-CoV-2-Infektion stark veränderte. Ribavirin hemmt das Enzym Inosinmonophosphat-Dehydrogenase (IMPDH), welches limitierend für die Synthese des RNA-/DNA-Bausteins Guanosin ist. Auch hier konnten wir in der Zellkultur zeigen, dass durch Einsatz von Ribavirin die Replikation von SARS-CoV-2 gehemmt wird (**Abb. 3B**). Die bereits erfolgte Zulassung zur Therapie anderer Erkrankungen, wie z. B. Hepatitis C, macht Ribavirin nun zu einem guten Kandidaten für weitere klinische Tests.

Generell zeigten weitere unserer Ergebnisse, dass während der Infektion mit SARS-CoV-2 zudem wachstumsanregende Signalwege aktiviert werden, die häufig auch bei Krebserkrankungen eine wichtige Rolle spielen [5]. Diese Signalwege reagieren auf Wachstumsfaktoren außerhalb der Zelle und regen Zellwachstum und Teilung an. Während dies in Krebserkrankungen zu unkontrollierter Zellteilung führt, ermöglicht es dem SARS-CoV-2-Virus während der Infektion sich selbst zu vermehren. Durch das jahrelange Studium dieser Signalwege im Kontext von Tumorerkrankungen finden sich bereits zahlreiche zugelassene oder in klinischen Tests befindliche Medikamente auf dem Markt. Mittels mehrerer dieser Medikamente (u. a. Sorafenib, Pictilisib und Omipalisib) konnten wir erfolgreich die Vermehrung von SARS-CoV-2 in Zellkultur verhindern.

### Fazit

Holistische Untersuchungen der Effekte von pathophysiologischen Veränderungen auf der molekularen und zellulären Ebene werden zunehmend wichtiger, um die zugrunde liegenden Prozesse zu verstehen und neue Behandlungsmöglichkeiten zu entwickeln. Mit dem Ausbruch eines neuen Virus – SARS-CoV-2 – konnte die Systembiologie dabei ihre Stärke ausspielen und schnell Einblicke in Wirtszellveränderungen und therapeutische Ansatzpunkte liefern. Zwar lassen sich die Ergebnisse aus Zellkulturexperimenten nicht direkt auf den menschlichen Organismus übertragen, sie liefern jedoch die Grundlagen zur weiteren Untersuchung in relevanten Modellsystemen und zur datenbasierten Auswahl von Wirkstoffen für klinische Studien. Und damit bringen sie uns ein ganzes Stück voran bei der Suche nach wirksamen Medikamenten gegen COVID-19.

## AUTOREN

**Kevin Klann**

**Christian Münch**

Hier steht
eine Anzeige.
Springer


Funding note

Open Access funding enabled and organized by Projekt DEAL.

Kevin Klann Jahrgang 1991. 2011–2014 Bachelor In Biowissenschaften an der Universität Frankfurt a. M. 2014–2016 Master in Molecular Biosciences an der Universität Heidelberg. Seit 2017 PhD-Student im Labor von Christian Münch am Institut für Biochemie II, Universitätsklinikum Frankfurt a. M. Spezialisiert in quantitativer Massenspektrometrie und Systembiologie.

Christian Münch Jahrgang 1982. Biochemiestudium an der Universität Tübingen. 2011 Promotion am MRC Laboratory of Molecular Biology and University of Cambridge, UK. 2012–2016 Postdoc an der Harvard Medical School, Boston, USA. Seit Ende 2016 Leiter einer Emmy-Noether-Forschungsgruppe und der Quantitativen Proteomikeinheit am Institut für Biochemie II des Fachbereichs Medizin an der Universität Frankfurt a. M.

Danksagung

Diese Arbeit wurde durch das Emmy-Noether-Programm der Deutschen Forschungsgemeinschaft (MU 4216/1-1) unterstützt.


Literatur

[1] Mohr I Sonenberg N Host translation at the nexus of infection and immunity Cell Host Microbe 2012 12 470 483 10.1016/j.chom.2012.09.006 23084916 PMC7104986
[2] Dauber B Wolff T Activation of the antiviral kinase PKR and viral countermeasures Viruses 2009 1 523 544 10.3390/v1030523 21994559 PMC3185532
[3] Hoehl S Berger A Kortenbusch M Evidence of SARS-CoV-2 infection in returning travelers from Wuhan, China N Engl J Med 2020 382 1278 1280 10.1056/NEJMc2001899 32069388 PMC7121749
[4] Bojkova D Klann K Koch B Proteomics of SARS-CoV-2-infected host cells reveals therapy targets Nature 2020 583 469 472 10.1038/s41586-020-2332-7 32408336 PMC7616921
[5] Klann K Bojkova D Tascher G Growth factor receptor signaling inhibition prevents SARS-CoV-2 replication Mol Cell 2020 80 164 174 10.1016/j.molcel.2020.08.006 32877642 PMC7418786
[6] Ong SE Blagoev B Kratchmarova I Stable isotope labeling by amino acids in cell culture, SILAC, as a simple and accurate approach to expression proteomics Mol Cell Proteomics 2002 1 376 386 10.1074/mcp.M200025-MCP200 12118079
[7] Weekes MP Tomasec P Huttlin EL Quantitative temporal viromics: an approach to investigate host-pathogen interaction Cell 2014 57 1460 1472 10.1016/j.cell.2014.04.028 PMC4048463 24906157
[8] Tatum EL Beadle GW Genetic control of biochemical reactions in neurospora: an “aminobenzoicless” mutant Proc Natl Acad Sci 1942 28 234 243 10.1073/pnas.28.6.234 16578042 PMC1078456
[9] Ideker T Krogan NJ Differential network biology Mol Syst Biol 2012 8 565 10.1038/msb.2011.99 22252388 PMC3296360
[10] Huttlin EL Bruckner RJ Paulo JA Architecture of the human interactome defines protein communities and disease networks Nature 2017 545 505 509 10.1038/nature22366 28514442 PMC5531611
[11] Klann K, Tascher G, Münch C (2021) Virus systems biology: proteomics profiling of dynamic protein networks during infection. In: Advances in Virus Research. Academic Press; doi:10.1016/bs.aivir.2020.12.00110.1016/bs.aivir.2020.12.001 33934824
[12] Martin P Jensen DM Ribavirin in the treatment of chronic hepatitis C J Gastroenterol Hepatol 2008 23 844 855 10.1111/j.1440-1746.2008.05398.x 18565019
[13] Marcelin JR Wilson JW Razonable RR Oral ribavirin therapy for respiratory syncytial virus infections in moderately to severely immunocompromised patients Transpl Infect Dis 2014 16 242 250 10.1111/tid.12194 24621016
